# AI-enhanced solutions during COVID-19: Current trends and future innovations

**DOI:** 10.1016/j.amsu.2022.104158

**Published:** 2022-07-13

**Authors:** Faisal A. Nawaz, Abdul Rahman Khan, Thomas Boillat

**Affiliations:** a College of Medicine, Mohammed Bin Rashid University of Medicine and Health Sciences, Dubai, United Arab Emirates; b Design Lab, College of Medicine, Mohammed Bin Rashid University of Medicine and Health Sciences, Dubai, United Arab Emirates

## 1 Introduction

**Fig. 1:**
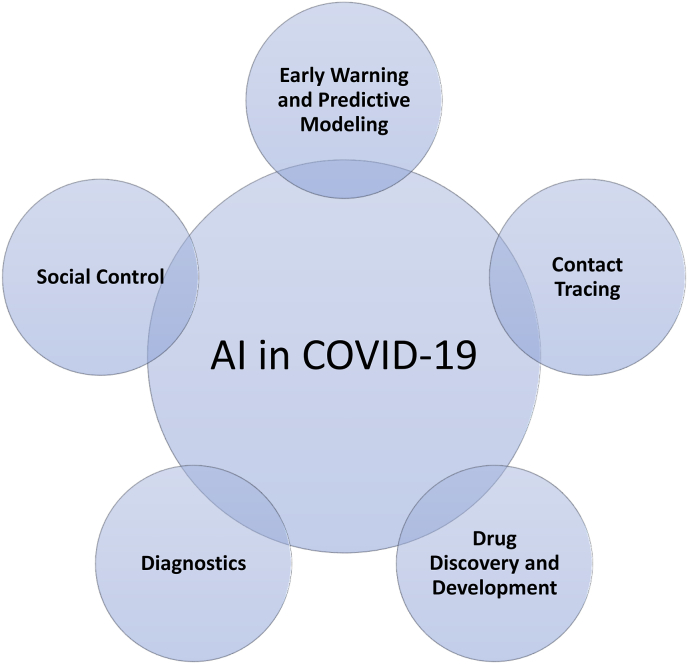
AI-assisted applications during the COVID-19 pandemic.

Artificial Intelligence (AI) is defined as a branch of Computer Science that is capable of simulating intelligent behavior through machine automation systems. There has been a significant rise in research and application of AI in addressing various aspects of Engineering and Medicine. This mutual overlap between the two fields has led to a new discipline, so-called “Artificial Intelligence in Medicine”, or AIM in short [1]. Not only is AIM being applied for image processing and analysis, but also for prognose [[2], [3], [4]], treatment [[5], [6], [7]], and patient monitoring [1,8] among others. In this matter, AIM has been instrumental during the Corona Virus Disease 2019 (COVID-19) pandemic. The Severe Acute Respiratory Syndrome Coronavirus 2 (SARS-CoV-2) has been brought to global attention and was declared a pandemic by the World Health Organization (WHO) on March 11, 2020 [9]. The exponential increase in the number of cases worldwide has prompted for emergent innovations and collaborations in the fields of Medicine and Engineering. While there have been contrasting opinions on the scope of AI during this period, we are observing a continuum of interdisciplinary growth across this field [10]. The early impact of AI during COVID-19 has been observed in 1) Early warning system and predictive modeling 2) Contact Tracing 3) Diagnostics 4) Drug discovery and development and 5) Social Control. As depicted in Fig. 1, this article aims to explore these domains [10] in the context of AI-assisted applications and their impact on addressing COVID-19. This article describes the contributions of AI during COVID-19, along with trends and innovations related to these technologies in harnessing sustainable healthcare solutions.

### 1.1 Early warning system and predictive modeling

Having an early warning system to detect outbreaks like COVID-19 can be deemed vital to the progression and management of population health on a global scale. BlueDot, an AI- based prediction model, had spotted what would come to be known as COVID-19, nine days before the World Health Organization [11]. As a collaborative effort between physicians, computer programmers and epidemiologists, this technology used natural language processing and machine learning to cull data from thousands of sources, including statements from official public health organizations, digital media, airline ticketing data, livestock health reports and population demographics.

While early warning models like BlueDot may help at the start of an outbreak, we are exploring similar innovation in predicting the course of this pandemic and the impact this has on the population curves. An AI-based model trained on past SARS dataset shows promise in the area of predictive modeling. In this context, SEIR is an epidemiological model used to predict disease dynamics by analyzing the population into four possible states: Susceptible [S], Exposed or latent (E), Infectious (I) or Removed (R) [12]. This model was highly effective in predicting the peaks and sizes of this outbreak based on different regions. This also helped study the varying changes in restrictions imposed on these populations, thereby giving deeper insights on the timing and strategies in optimally limiting the spread of COVID-19.

**Fig. 2:**
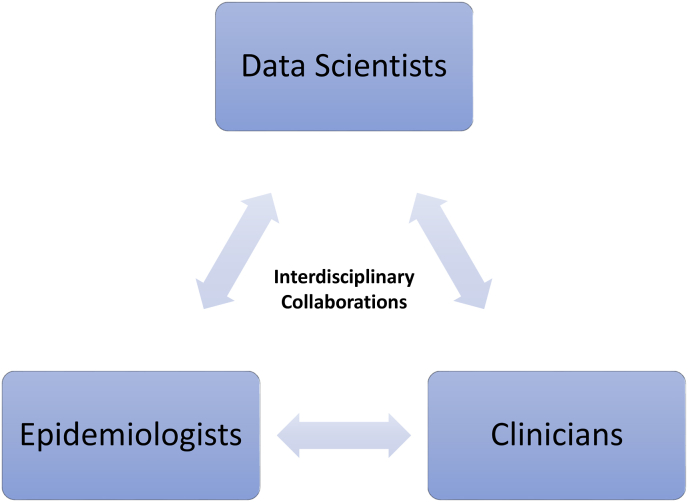
Triangle of collaborations for optimizing AI applications in healthcare.

This shared perspective brings to light on the importance of having data scientists on the same page as epidemiologists and clinicians. As shown in Fig. 2, this triangle of collaboration will help in optimizing the translation of early prototypes to global practice with efficiency. Even though the scope of this prediction was limited to being 9 days early, AI can be enhanced to make greater leaps in predicting future pandemics and devising the ideal prevention strategies based on larger populations. Understanding the functionality of such complex neural networks is still lacking in today's field and closing this essential knowledge gap could be the answer to better development and implementation of this promising technology.

### 1.2 Drug discovery and development

The role of Artificial Intelligence in Drug discovery and development has been one of the most sought-after innovations in this race against COVID-19. While there are various drugs currently in clinical trials with limited outcomes so far, we are yet to find the ideal solution for treatment in this fast-paced pandemic. AI can help with simulating potential drug combinations on a massive scale and analyzing the viral genome for potential drug targets at the same time. A company called Exscientia [13] is combing through a library of 15,000 molecules with AI to understand the best possible treatment approach against this virus. This amount of data processing could be a virtual game-changer when manual exploration has taken much longer to reach the same conclusions. Another approach was implemented by an AI-based technology called BenevolentAI, where they offer interactive visualizations of the connections among diseases, symptoms, and biological processes, sourced from databases and machine-learning algorithms that process text in scientific papers. This led to the early finding of baricitinib (used to treat rheumatoid arthritis) as a potential re-purposed drug against COVID-19 [14].

Understanding the impact of Biomedical engineering in drug development assisted by AI may be one of the most powerful interventions in containing this pandemic. The applications mentioned so far are limited by current research on understanding the genomic structure of this virus. Increased funding is required along with improved regulations for expediting the process of drug development to determine a clinically impactful solution at the earliest.

### 1.3 Contact tracing

A solution that is increasingly being relied upon and actively being used in multiple countries is COVID-19 contact tracing using smartphone technology [15]. Contact tracing can help the individuals understand their risk and limit further spread of the virus. Contact tracing as an epidemic control measure is not novel; in fact it is one of the first-line measures to combat highly infectious diseases, and has been deployed against other illnesses such as measles, SARs, typhoid, meningococcal disease and sexually transmitted infections like AIDS. The use of smartphone technologies and various other technologies to help identify and trace individuals with various diseases had previously been proposed during the Ebola epidemic [16]. With the democratization and capacity of smartphones, the potential of such technology increases constantly. Why would such apps be useful at all? Emerging research shows that a major proportion of people with COVID-19 could be asymptomatic and spread it rapidly within a population unknowingly [17]. Therefore, only quarantining people after they show symptoms might not be effective to control this pandemic. Public health authorities would need to act quickly when a patient is diagnosed with COVID-19 to find all the people he/she was in close proximity with. Mathematical models of the pandemic [18] show that rapid efficient contact tracing combined with a large-scale virus screening program could be highly effective in controlling this pandemic. However, only a digital, automated tool using bluetooth or location tracking technology could make such fast contract tracing possible. If a phone has been in close proximity to an “infected” phone, the user of that phone will receive a notification to immediately go into self-quarantine at home and the relevant health authorities will be notified. Examples of the application of such contact tracing technology include the Al-hosn app which is being used in the United Arab Emirates to trace COVID-19 contacts [19].

As essential and effective these technologies are, the fear or limitation is that, once the pandemic ends, this “invasion” of data privacy may not be entirely rolled back by certain governments or institutions who would continue to use this tested ability to conduct large scale surveillance over the population and use the COVID-19 data for other purposes. Some countries such as Switzerland are guaranteeing privacy protection to users as the data is only saved locally and neither the mobile phone nor the app sends any personal or location data to a central storage location or server [20].

### 1.4 Diagnostics and prognostics

Other than predictive modeling and contact tracing, perhaps one of the most fundamental applications of AI is in diagnostics and prognostics. Rapid and accurate diagnosis of COVID-19 has a potential to not only save the patients’ lives but also help in controlling this pandemic. There is emerging research and work currently being done in training AI models to accurately diagnose COVID-19 cases using chest X-rays and CT scans. According to a recent review of AI applications against COVID-19 [10], AI can be equally if not more accurate than humans, can reduce the burden on radiologists, and perform a diagnosis faster and cheaper than standard COVID-19 testing. This would need the valuable input of a wide range of technical experts including biomedical engineers, computer programmers and software engineers to make sure an accurate and efficient diagnostic tool is created.

Wang and Wong [12] proposed a deep model for COVID19 detection (COVID-Net), which obtained 92.4% accuracy in classifying normal, non-COVID pneumonia, and COVID-19 classes. In addition, there are several recent studies on COVID-19 diagnostics using deep learning models on CT scans [21].

Since most COVID-19 patients do not require intensive care treatment, if we were able to accurately predict or forecast some of the debilitating or severe manifestations of the disease, that would be extremely useful to the healthcare system as it could help with resource allocation and reducing the burden.

At a massive scale, the application of AI in diagnostics and prognostics has not been in widespread practice with concerns ranging from that there isn't enough data available to train AI models since most of these images come from Chinese hospitals and this could lead to a selection bias, to the increased risk of equipment contamination. Secondly, one of the most important disadvantages of chest radiography analyses is an inability to detect the early stages of COVID-19, as they do not have sufficient sensitivity in ground-glass opacification detection [22].

### 1.5 Social control

Another important role of AI in managing this pandemic is social control. In the last few weeks, AI has increasingly been used by countries globally for thermal imaging to detect potentially infected individuals and also to enforce lockdown and social distancing measures [23]. Thermal imaging however has a major limitation as it cannot differentiate whether a person has symptoms from COVID-19 or some other underlying illness [24]. Unmanned Aerial Vehicles (UAVs), commonly known as drones, are also becoming vital tools in the fight against COVID-19 as they have increasingly been used by law enforcement agencies to monitor public gatherings, ensuring social distancing, spraying disinfectants and overseeing cargo.

AI is also being used to enforce self-quarantine orders using facial-recognition and location tracking technology. A computer vision software company based in the US is already offering a “social distancing breach detection” tool which can monitor social distancing protocols and warn the authorities when breached [25]. Similar solutions are also being explored and tested in other parts of the world like the UK and India [26,27]. According to Petropoulos [28] such apps can “enable patients to receive real-time waiting-time information from their medical providers, to provide people with advice and updates about their medical condition without them having to visit a hospital in person, and to notify individuals of potential infection hotspots in real-time so those areas can be avoided”.

These technologies would require the expertise of mechanical, software and computer engineers as well as highly capable cyber-security experts since the information collected could be quite sensitive in terms of data privacy and confidentiality.

## 2 Conclusion

Artificial Intelligence in healthcare has unraveled a potential for public health innovation during the COVID-19 pandemic. Various AI-based interventions have been applied in the general community through collaborative efforts. Understanding the current advancements and their limitations is vital to predicting and trusting any foreseeable trends with AI-powered solutions. Future research is warranted in exploring the scalability of such solutions to manage future pandemics and allow for strategic preparedness against rising COVID-19 variants amidst other global outbreaks.

## Ethical approval

N/A.

## Sources of funding

This work was funded by 10.13039/501100020917Mohammed Bin Rashid University of Medicine and Health Sciences (MBRU).

## Author contributions

All individuals who meet authorship criteria are listed as co-authors who have participated adequately in this work to take public responsibility for the generated results and content, including participation in the idea, design, writing of the manuscript, or revision. Furthermore, each author listed certifies that this material or similar material has not been and will not be submitted to or published in any other publication before its appearance in **The Annals of Medicine and Surgery.**

Conceptualization: FN, AK, TB; Writing original draft: FN, AK; Review and editing: FN, AK, TB.

## Registration of research studies

1. Name of the registry: N/A.
2. Unique Identifying number or registration ID: N/A.
3. Hyperlink to your specific registration (must be publicly accessible and will be checked): N/A

## Guarantor

N/A.

## Consent

N/A.

## Declaration of competing interest

None declared.
